# Evaluation of Clipping Based Iterative PAPR Reduction Techniques for FBMC Systems

**DOI:** 10.1155/2014/841680

**Published:** 2014-01-16

**Authors:** Zsolt Kollár, Lajos Varga, Bálint Horváth, Péter Bakki, János Bitó

**Affiliations:** ^1^Inter-University Centre for Telecommunications and Informatics (ETIK), Kassai straße 26, Debrecen H-4028, Hungary; ^2^Department of Broadband Infocommunications and Electromagnetic Theory, Budapest University of Technology and Economics (BME), Egry József utca 18, Budapest H-1111, Hungary

## Abstract

This paper investigates filter bankmulticarrier (FBMC), a multicarrier modulation technique exhibiting an extremely low adjacent channel leakage ratio (ACLR) compared to conventional orthogonal frequency division multiplexing (OFDM) technique. The low ACLR of the transmitted FBMC signal makes it especially favorable in cognitive radio applications, where strict requirements are posed on out-of-band radiation. Large dynamic range resulting in high peak-to-average power ratio (PAPR) is characteristic of all sorts of multicarrier signals. The advantageous spectral properties of the high-PAPR FBMC signal are significantly degraded if nonlinearities are present in the transceiver chain. Spectral regrowth may appear, causing harmful interference in the neighboring frequency bands. This paper presents novel clipping based PAPR reduction techniques, evaluated and compared by simulations and measurements, with an emphasis on spectral aspects. The paper gives an overall comparison of PAPR reduction techniques, focusing on the reduction of the dynamic range of FBMC signals without increasing out-of-band radiation. An overview is presented on transmitter oriented techniques employing baseband clipping, which can maintain the system performance with a desired bit error rate (BER).

## 1. Introduction

Following the digital switchover, a significant amount of spectra remain unexploited in frequencies previously licensed for analogue television broadcast due to the high spectral efficiency of digital techniques [1]. Cognitive radio is a method for reaggregating unused or partially used spectral sections called white spaces and gray spaces [2]. Cognitive radios are smart, adaptive, and opportunistic systems equipped with special spectral sensing capabilities. These opportunistic devices must not cause interference for the incumbent users of the spectrum, who own the primary service allocated to the given frequencies. To meet this criterion, strict spectral masks are defined for the transmit signal by various regulators such as Ofcom (UK) and FCC (US). These regulators have requirements on ACLR level of at least −55 dB in the neighboring bands [3, 4].

OFDM using cyclic prefix (CP-OFDM) is one of the most commonly applied and widespread modulation schemes, used in many communication standards such as DVB, DAB, LTE, and others. However, OFDM shows some critical drawbacks in certain usage scenarios. Most importantly, the ACLR of CP-OFDM fails to meet the requirements of the spectral mask introduced for cognitive radio. Two approaches may be taken to overcome this issue: additional time domain filtering can be applied [5] or some subcarriers may be reserved for the reduction of out-of-band radiation [6]. Both methods have negative side-effects: the former may cause performance degradation and the latter results in high computational load and data rate reduction. The ACLR level may also be elevated by distortion introduced by nonlinearities in the transceiver chain.

Various alternative multicarrier modulation schemes have emerged for cognitive use instead of OFDM. The suitability of some alternative schemes has been investigated in [7]. The most promising scheme—with extremely low ACLR—is FBMC [8], also known as staggered multitone (SMT) [9] or OFDM with Offset QAM (OFDM/OQAM) [10].

With a large number of subcarriers, FBMC signals show time domain characteristics similar to OFDM. The amplitude distribution of the transmit signal is Gaussian, which leads to high PAPR. High PAPR is especially problematic with nonlinearities, as the nonlinear effect is more pronounced for signals of high dynamic range. To minimize the effects of nonlinearities while maintaining the efficiency of the power amplifier, one must reduce the PAPR with a method causing no significant performance loss in data transmission and/or no regrowth of out-of-band radiation.

Numerous PAPR reduction techniques have been presented for OFDM in [11]. These techniques are not directly applicable to FBMC, as FBMC symbols are overlapping in the time domain; therefore symbol-by-symbol PAPR reduction can not be performed. PAPR reduction methods specifically for FBMC systems have already been proposed in the literature:

- reducing the number of subcarriers [12],
- clipping with filtering (CF) and receiver oriented compensation [13],
- selective mapping (SLM) [14],
- single carrier frequency division multiple access FBMC (SC-FDMA FBMC) [15].

Each method has its own drawbacks: reducing the number of subcarriers reduces data rate, CF requires a modification of the receiver to restore the performance and moves most of the signal processing to the receiver side, SLM requires the transmission of additional side information for the demodulation, and SC-FDMA FBMC shows only moderate PAPR reduction performance.

This paper introduces and investigates novel clipping based iterative PAPR reduction techniques exploiting the *Bussgang theorem* [16], which can be applied on the transmitter side of FBMC systems. The techniques do not impose modifications to the receiver in order to maintain overall system performance. The advantageous spectral properties of the transmitted signal also remain unaltered. The paper also introduces novel metrics for evaluation of iteration performance of PAPR reduction in FBMC systems.

## 2. FBMC

### 2.1. System Model

**Figure 1:**
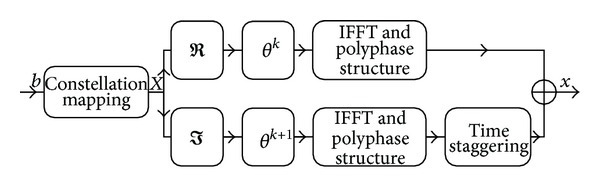
Block diagram of FBMC transmitter.

FBMC is a class of multicarrier modulation schemes with a modulated prototype filter *p*_0_(*t*) applied for each subcarrier. The prototype filter fulfils the Nyquist property [10]. Due to the advantageous properties of the prototype filter, the FBMC signal has better spectral efficiency compared to OFDM, where a rectangular window is applied on the subcarriers [9]. Detailed investigation on the performance of various prototype filters is given in [18]. The details of FBMC signal generation can be seen in Figure 1 [19, 20]. The procedure consists of the following steps: binary information *b* is mapped using the complex modulation alphabet *𝒜* (a QAM set with Gray mapping), where each modulation symbol *X* represents *M* bits using offset-QAM modulation on the subcarriers. The real (*ℜ*) and imaginary (*ℑ*) parts of the complex FBMC modulation symbol *X* are transmitted with a time offset of half a symbol duration. To maintain the orthogonality of the filters the application of cyclic prefix should be avoided in FBMC systems. The modulated FBMC signal using *N* subcarriers and a discrete (sampled) prototype filter *p*_0_[*n*] can be expressed as

(1) x[n]=∑m=−∞∞∑k=0N−1(θkℜ{Xk[m]}p0[n−mN]      +θk+1ℑ{Xk[m]}p0[n−mN−N2]) ×ej(2π/N)k(n−mN),

where $j=\sqrt{-1}$, *θ* ^*k*^ = *e* ^*j*(*π*/2)*k*^ and *X*_*k*_[*m*] represent the modulation symbol on the *k*th subcarrier in the *m*th signalling time. As a consequence of the length of the filters' impulse response and the effort to neglect data rate loss the transmitted symbols overlap in the time domain, but a special filter construction enables the separation of symbols at the receiver. The overlapping ratio *K* of the consecutive symbols depends on the length of the prototype filter. The filter is designed with an impulse response of length *L* = *KN*, meaning that the symbol duration is stretched but with overlapping the original data rate is maintained. Throughout this paper the prototype filter described in [21] is applied with an overlapping factor of *K* = 4. Equation (1) can be implemented in a computationally efficient way using IFFT and a polyphase decomposition of the modulated prototype filters, with the two output signals being time staggered and added as presented in [22, 23].

### 2.2. Signal Statistics

#### 2.2.1. Dynamic Properties

**Figure 2:**
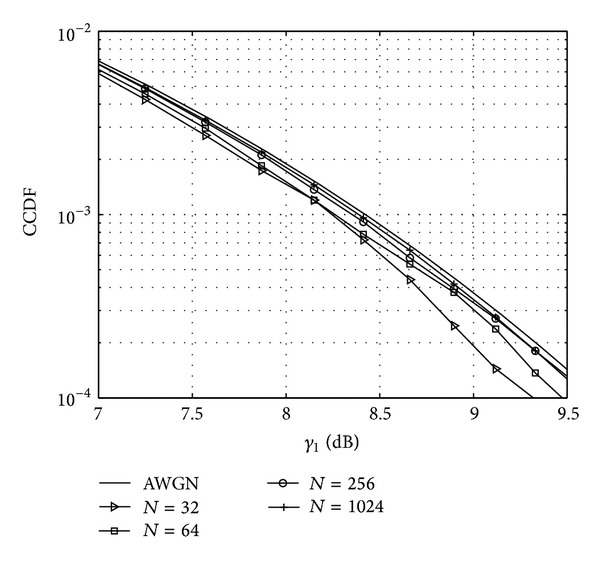
CCDF of the FBMC signal as a function of the number of subcarriers.

To describe the transmission signal's dynamic range the complementary cumulative distribution Function (CCDF) can be calculated for the signal energy or for the PAPR. Signal energy may be characterized by the following expression:

(2) $$\begin{array}{c} \gamma_{1}\left[n\right]=\frac{{\left|x\left[n\right]\right|}^{2}}{P_{0}}, \end{array}$$

where |*x*[*n*]| is the absolute value of the magnitude of the *n*th sample and *P*_0_ is the average power of the transmitted signal. On Figure 2 the CCDF for *γ*_1_ is presented for FBMC signals at different number of subcarriers. With increasing number of subcarriers the FBMC signal tends to be Gaussian distributed. This is in line with the *central limit theorem*, as having more subcarriers means more independent random variables being summed up.

The investigation of dynamic properties of FBMC signals can be performed blockwise. In this case only the highest magnitude value is extracted to characterize the entire block of samples. This metric is known as PAPR. The PAPR can be calculated for the *m*th symbol as

(3) $$\begin{array}{c} \gamma_{L}\left[m\right]=\frac{\max_{n}\left\{{\left|x\left[n\right]\right|}^{2}\right\}}{P_{m}},\\ n\in \left[\left(m-1\right)N,m\left(L+\frac{N}{2}-1\right)\right], \end{array}$$

where *P*_*m*_ is the average power of the *m*th symbol. PAPR can be expressed with *γ*_*L*_ as

(4) $$\begin{array}{c} \text{PAPR}{\left(\gamma_{L}\left[m\right]\right)}_{\text{dB}}=10\;\log_{10}\left(\gamma_{L}\left[m\right]\right). \end{array}$$

**Figure 3:**
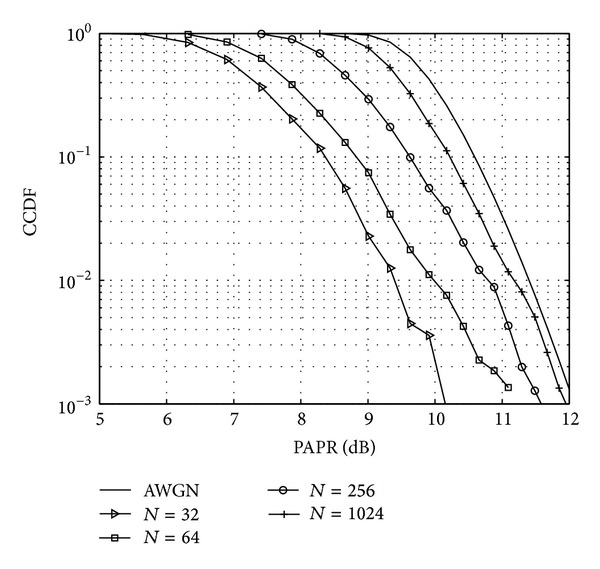
CCDF of the PAPR values of the FBMC symbols as a function of the number of subcarriers.

The CCDFs of the simulated PAPR values for different number of subcarriers are presented in Figure 3. It can be observed on both Figures 2 and 3 that, with increasing number of subcarriers, the CCDF curve of both metrics gets closer to the theoretical CCDF of the normal distribution. These figures are results of simulations using 10000 FBMC symbols with 16-QAM modulation.

#### 2.2.2. Kurtosis

**Figure 4:**
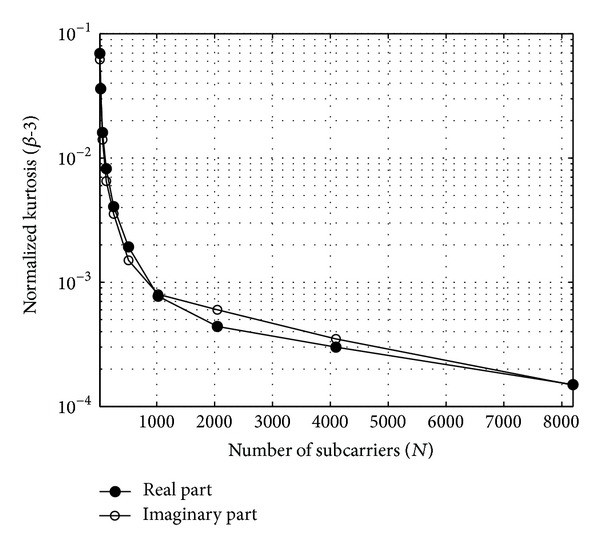
Normalized kurtosis (*β*-3) versus subcarrier number (*N*) of the FBMC signal using 16-QAM.

Kurtosis is a measure to statistically describe the shape of a distribution and its relation to the normal distribution. The kurtosis (parameterized with *β*) of the random variable *ξ* will be employed in the following discussion. Parameter *β* of *ξ* is commonly defined as

(5) $$\begin{array}{c} \beta =\frac{E\left\{\xi^{4}\right\}}{{\left[E\left\{\xi^{2}\right\}\right]}^{2}}=\frac{\mu_{4}}{\sigma_{s}^{4}}; \end{array}$$

that is, the kurtosis is the ratio between the signal's fourth moment *μ*_4_ and the square of its average power *P*_*s*_^2^ = (*σ*_*s*_^2^)^2^. Please note that *β* = 3 for signals having Gaussian distribution. The normalized kurtosis of the real and imaginary parts of the simulated FBMC signal can be seen in Figure 4. It can be observed that with increasing number of subcarriers the kurtosis of the signal is approaching that of Gaussian distribution.

#### 2.2.3. Power Spectral Density (PSD)

**Figure 5:**
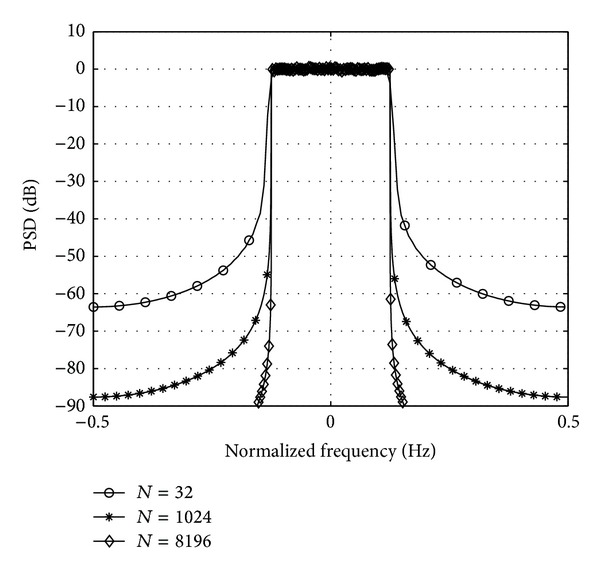
PSD of the FBMC signal as a function of the number of subcarriers (*N*).

For cognitive radio systems strict requirements are posed on ACLR. FBMC systems using prototype filters can implicitly fulfil these requirements. In Figure 5 the PSD of an FBMC signal with a prototype filter shown in [21] can be seen as a function of the number of subcarriers. In the simulations all available subcarriers were modulated using 16-QAM and an oversampling ratio of 4 was applied. Please note that the posed out-of-band radiation can almost be achieved with 32 subcarriers. An increase in subcarrier number gives further improvement in ACLR. However, increasing the number of subcarriers also increases the PAPR, as depicted in Figure 3. This means that a signal with higher number of subcarriers is more prone to nonlinear distortions, as the higher dynamic range may push the analogue components into their nonlinear range. For the effects of nonlinearity on the PSD of FBMC signals please refer to [7, 24].

## 3. Clipping Based PAPR Reduction Techniques for FBMC

### 3.1. Clipping Based Techniques

This section focuses on transmitter side methods for transmitting signal PAPR reduction. The targeted methods operate with unmodified receiver structure, enabling PAPR reduction without significant ACLR and BER performance degradation. PAPR reduction is also desired in system identification, where multisines (similar as those used in wireless communication systems) are commonly used measurement signals [25]. While in measurement systems the amplitude and phase of the sines are used for evaluation purposes, wireless communication uses the amplitude and phase as data carrying entities. A clipping aided iterative PAPR reduction scheme for multisines has already been developed for use in system identification scenarios. The scheme is presented in [26]. Clipping introduces hard nonlinear distortion, which has a significant effect on the overall BER performance [27]. To overcome this phenomenon, repeated clipping and filtering (introduced in [28]) can be applied for OFDM. Using this method the PAPR can be reduced significantly, but at the same time a considerable amount of nonlinear distortion is introduced. Based on the aforementioned ideas and incorporating their advantages, this section presents PAPR-reduction algorithms suitable for FBMC.

First, a simple clipping and filtering technique is presented for FBMC signals; then two techniques are discussed for which some additional signal processing is required. Tone reservation (TR) [29] allocates specific subcarriers for PAPR reduction purposes, leading to a loss of data rate. Active constellation extension (ACE) [30] allows the outer constellation points to be enlarged dynamically (in position). A possible combination of the two schemes is also presented later in the section. Please note that the negative side-effect of both techniques is an increased mean signal power. It is important to mention that both techniques are incorporated for OFDM in the DVB-T2 standard [31].

**Figure 6:**
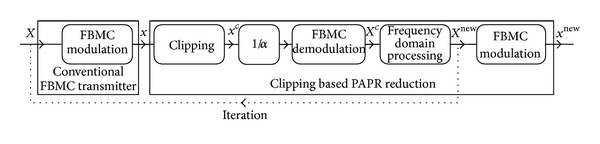
Block diagram of the clipping based PAPR reduction schemes.

**Figure 7:**
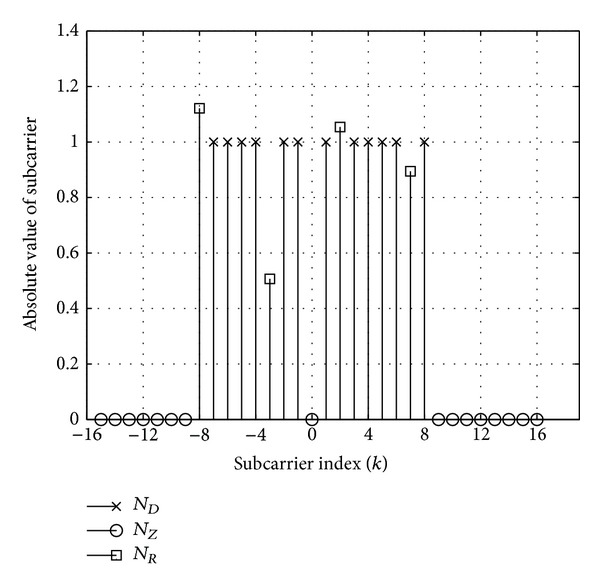
Subcarrier index notation.

Clipping based PAPR reduction schemes presented in this paper have common execution steps. In fact, these techniques can be represented using a common block diagram, shown in Figure 6. The only difference of the methods is the implementation of frequency domain processing. According to the block diagram the *X*_*k*_ data symbols are used to synthesize an FBMC symbol, where *k* represents the subcarrier index. Each symbol consists of *N* subcarriers in two subsets: *N*_*D*_ represents the subcarrier indices used for data transmission and *N*_*Z*_ denotes the indices of the unused (zero-valued) band-edge and DC subcarriers. The index notation of the various subcarriers is depicted in Figure 7. The subcarriers marked by *N*_*R*_ are special reserved carriers and their role will be detailed later.

A time domain clipping is performed on the signal after modulation. A mathematical description of the clipping procedure can be given as

(6) xc[n]={x[n],if  |x[n]|≤AmaxAmaxejφ(x[n]),if  |x[n]|>Amax,

where *x* ^*c*^[*n*] is the clipped signal, *x*[*n*] is the original signal, and *φ*(*x*[*n*]) is the phase of the original complex signal. The maximum magnitude of the clipped signal is defined by the clipping ratio (CR)

(7) $$\begin{array}{c} \text{C}{\text{R}}_{\text{dB}}=10\;\log_{10}\left(\gamma_{c}\right), \end{array}$$

with *γ*_*c*_ = *A*_max_^2^/*P*_0_. The mathematical model of the clipped signal can be derived from the *Bussgang theorem* [16] and it is given in the following form:

(8) $$\begin{array}{c} x^{c}\left[n\right]=\alpha x\left[n\right]+d\left[n\right], \end{array}$$

where *α* is an attenuation factor and *d*[*n*] is the clipping noise which is uncorrelated with the signal *x*[*n*]. The attenuation factor *α* can be calculated as a function of the clipping ratio *γ*_*c*_ as

(9) $$\begin{array}{c} \alpha =1-e^{-\gamma_{c}}+\frac{\sqrt{\pi }}{2}\sqrt{\gamma_{c}}\;\mathrm{erfc}\left(\sqrt{\gamma_{c}}\right). \end{array}$$

Clipping decreases the signal power and this has to be compensated with a multiplication of 1/*α*. After clipping and magnitude compensation the signal is demodulated, giving data symbols *X*_*k*_^*c*^. These symbols are used as inputs of the frequency domain processing block, which runs the PAPR reduction algorithm. The detailed description of the methods concerned will be presented later in this section. Following the processing, the new data symbols *X*_*k*_^new^ are obtained, which are then used for FBMC modulation. This modulated signal can be either transmitted or fed back to the clipping stage, thus giving an iterative realization. All clipping based methods can be performed iteratively, leading to reduced PAPR values. The optimal number of iterations will be discussed in Section 4.

**Figure 8:**
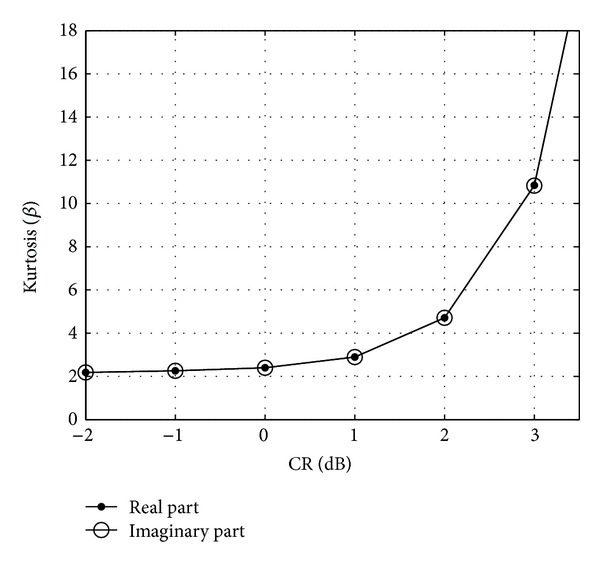
Kurtosis (*β*) of the clipping noise as a function of the clipping ratio (CR).

Besides the reduction of PAPR, another main goal of each method is to preserve the FBMC signal's favourable ACLR and BER properties. For the simulations a clipping ratio of 1 dB is preferred, where the distribution of the clipping noise *d* is the closest to Gaussian. This can be seen in Figure 8, where the kurtosis of the real and imaginary parts of the clipping noise is presented as a function of the CR. For low and moderate clipping ratios: 0 dB ≤ CR ≤ 3 dB the clipping noise can be assumed to be Gaussian. The choice of CR below 0 dB is not reasonable in real life applications.

In the following subsections the applied PAPR reduction techniques are introduced with a description of their advantages and disadvantages.

#### 3.1.1. Clipping and Filtering (CF)

The idea of this method was introduced in [28] for OFDM signals. After clipping and demodulation the *X*_*k*_^*c*^ data symbols with index *k* ∈ *N*_*Z*_ are reset to their original values (i.e. 0 + *j*0), with all other subcarriers left intact. The resulting *X*_*k*_^new^ are then used for FBMC modulation. The advantage of this method is its low complexity; it also leads to the lowest PAPR. However, due to the clipping noise, this scheme undesirably degrades BER. The PAPR results achieved by this method can serve as a reference targeted by the forthcoming algorithms. Please note that receiver oriented clipping noise mitigation techniques are available to improve the BER performance, as presented in [13, 17]. The most significant drawback of these techniques is the demand for computationally intensive calculations.

#### 3.1.2. Tone Reservation (TR)

This scheme was originally introduced for OFDM signals in [29]. The main idea is to have a set of subcarriers *N*_*R*_ (i.e., reserved tones), which can have arbitrary values after clipping and demodulation. The processing in this case is as follows. The clipped and demodulated *X*_*k*_^*c*^ data symbols for data carrying subcarriers *k* ∈ *N*_*D*_ are reset to their original values. The demodulated symbols for *k* ∈ *N*_*R*_ remain unchanged. This means that *X*_*k*_^new^ will take the following values:

(10) Xknew={0+j0,for  k∈NZ,Xk,for  k∈ND,Xkc,for  k∈NR.

The more subcarriers are reserved, the lower PAPR can be achieved. Increasing the number of reserved subcarriers has a practical limit, as reserved tones are unavailable for data transmission. A balance is desired between data rate loss and PAPR reduction. The advantages of this method include a low complexity and an unaltered BER, as the data carriers are not affected by the method.

#### 3.1.3. Active Constellation Extension (ACE)

**Figure 9:**
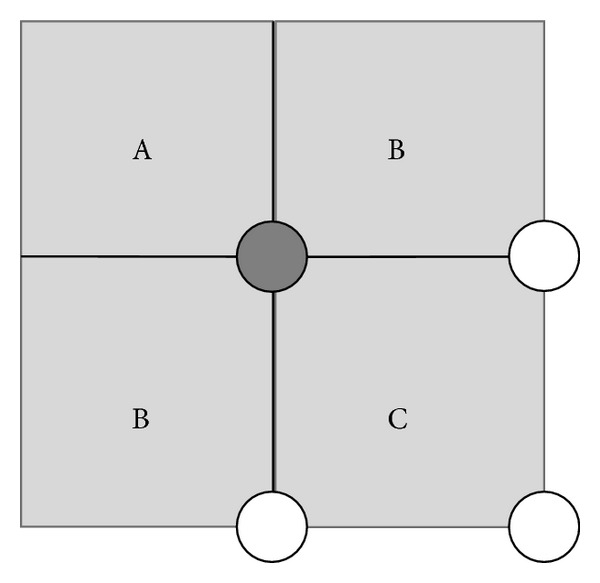
Constellation diagram and ACE decision regions for the marked symbol of 4-QAM.

**Figure 10:**
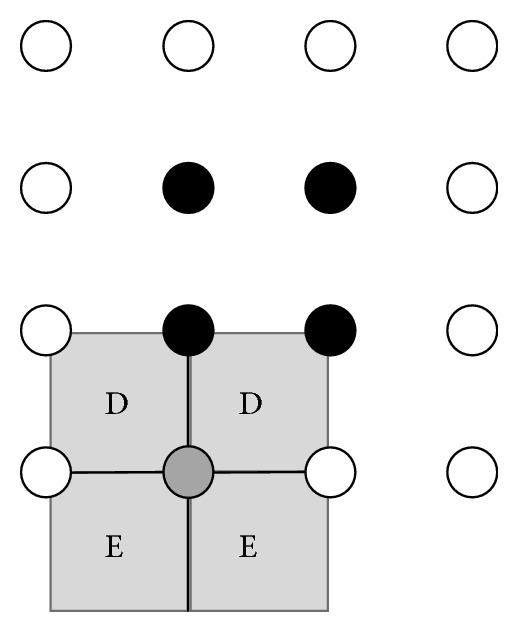
Constellation diagram and ACE decision regions for the marked symbol of 16-QAM.

The basics of this method were investigated for OFDM signals in [30]. The basic concept is that after clipping and demodulation the data symbols' value (i.e., the constellation points' position) can be altered in a way that the Euclidian distance between the constellation points is increased. In the case of *X*_*k*_^new^ for indices *k* ∈ *N*_*Z*_ the values are reset to 0. The data symbols may either be reset to their original value *X*_*k*_, retain the new (clipped) value *X*_*k*_^*c*^, or obtain the new value with a mapping algorithm. During this mapping algorithm a projection method is used, which is demonstrated for 4-QAM and 16-QAM in Figures 9 and 10, respectively.

In case of 4-QAM the values *X*_*k*_^*c*^ originating from the constellation point marked by gray color may fall in the following regions:

- *A*: values which fall in this region remain unaltered.
- *B*: the values are orthogonally projected onto the borderlines of the regions *B* and *A*.
- *C*: values in this region are reset to their original values *X*_*k*_.

For 16-QAM the same rules apply to the four corner constellation points as for 4-QAM. If the clipped symbols originate from the internal constellation symbols marked with black color: they are reset to their original values. For the values *X*_*k*_^*c*^ originating from the kind of side constellation points marked by gray color in Figure 10 (i.e., symbol constellation border but not corner points) may fall in the following regions:

- *D*: values falling in this region are reset to their original values *X*_*k*_.
- *E*: the values are orthogonally projected onto the borderline of the two *E* regions.

Further alternative techniques for the processing of the clipped symbols can be found in [30]. This method reduces PAPR more effectively than the TR scheme while still retaining the BER. On the other hand, this method has a high computational complexity; furthermore it significantly increases the average power of the signal and prevents the use of a soft decision based demodulation in the receiver.

#### 3.1.4. Joint Use of TR and ACE (TRACE)

Since TR and ACE methods may be operated on different subcarriers (having indexes *N*_*R*_, *N*_*D*_), these methods can be applied simultaneously. The joint application of these methods may lead to further improved PAPR reduction performance. However, their simultaneous application also combines their disadvantages and raises the computational complexity at the same time. A similar idea using a signal model slightly different from (8) for OFDM was presented in [32].

## 4. Implementation Aspects

This section discusses the implementation aspects of the presented iterative PAPR reduction schemes. The discussion starts with the computational complexity of the proposed schemes and continues with the formalization of the effects of the different parameters on PAPR reduction performance.

### 4.1. Computational Complexity of the Different Methods

This section discusses the computation requirements of the previously presented PAPR reduction techniques. As seen in [33], CF provides the best PAPR performance. If CF is used without iteration, receivers employing iterative decoding (as presented in [13, 34]) can compensate for the resulting BER degradation. Further PAPR reduction can be obtained using CF in an iterative manner, as described in [28]. In this case bit errors are introduced by the nonlinear distortion terms, which cannot be compensated for. CF only requires FBMC modulation, clipping, and FBMC demodulation blocks at the transmitter side. Subcarriers with indices *k* ∈ *N*_*Z*_ are reset to 0.

The complexity of the TR method is the same as that of CF; however, the performance is strongly dependent on the number of reserved tones. The subcarriers with indexes *k* ∈ *N*_*Z*_ are reset to 0, the data subcarriers *k* ∈ *N*_*D*_ are restored to their original values, and the value of the reserved tones *k* ∈ *N*_*R*_ remains unaltered.

The ACE method has a slightly higher computational complexity due to the procedure of mapping of the distorted constellation point on the data subcarriers as presented in Section 3.1.3. The zero subcarriers have to be reset to 0 as well.

Most of the signal processing complexity of the iterative PAPR reduction technique presented in Figure 6 goes to the modulation and demodulation of the FBMC signal. Iterative modulation, clipping, and demodulation can be performed on an FBMC signal burst but this is very time-consuming. Due to the overlapping nature of the FBMC signal, after a delay of *L* = *KN* samples, the clipped symbols can be demodulated and after the frequency domain preprocessing the modulation can be restarted in a parallel manner, on a parallel thread. This implementation can be especially efficient in FPGA with a predefined number of iterations. A computationally efficient modulation with low complexity for FBMC can be found in [35]; for continuous and fast demodulation of the FBMC symbols the recursive discrete Fourier transform can be applied as presented in [36].

### 4.2. Optimal Iteration Number and Clipping Ratio

Two key parameters for the PAPR reduction method are the number of iterations and the clipping ratio. In this paper the clipping ratio is considered to be fixed during iterations. Further investigations should be performed to find the best clipping ratio profile as a function of iterations.

To characterize the gain of the PAPR reduction method a metric is defined which corresponds to the PAPR reduction performance and the invested additional signal power. The PAPR gain is defined as

(11) Θ=maxm{PAPR(x[m])dB}−maxm{PAPR(xnew[m])dB}+ΔPs,dB,

where PAPR(*x*[*m*])_dB_ is the original signal's highest PAPR, PAPR(*x* ^new^[*m*])_dB_ is the highest PAPR value of the modified signal *x* ^new^, and Δ*P*_*s*,dB_ is the ratio of the average power of the original signal *x* and the modified signal *x* ^new^ in dB. The metric takes into account the PAPR gain and increased transmit power. As a result, if the peak of the signal remains unchanged but the signal power is enlarged it has no contribution to the PAPR reduction as the PAPR reduced signal is scaled to the maximal linear range of the amplifier, so it will not lead to any additional increase in the output power. For TR this can be interpreted as only the power on the reserved tones being enlarged; thus no further gain can be achieved. During the simulation this metric will also be investigated.

## 5. Simulations

### 5.1. PAPR Performance

This section presents simulation results for the introduced PAPR reduction schemes. The techniques are evaluated based on the CCDF of the PAPR as a function of the iterations. The gain metric Θ (presented in (11)) is also compared for each PAPR reduction technique. For each method, the optimal number of iterations was determined based on the evolution of the gain metric. After a specific number of iterations, the value Θ begins to decrease; from this point on further iterations are not providing any additional gain. An iteration number of 5 was selected for ACE and TRACE and an iteration number of 15 was chosen in case of TR. The simulation parameters for the FBMC signal and for the PAPR reduction methods are summarized in Table 1. The FBMC prototype filter is the one defined in [21] with an overlapping factor of *K* = 4.

**Figure 11:**
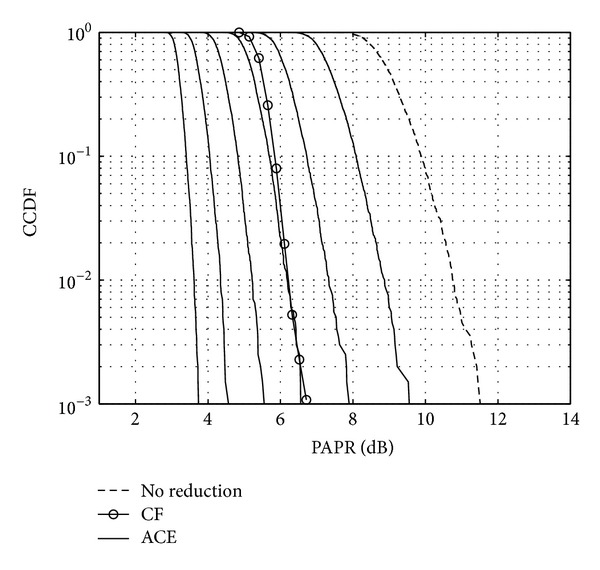
CCDF of the PAPR values using ACE reduction iteratively.

**Figure 12:**
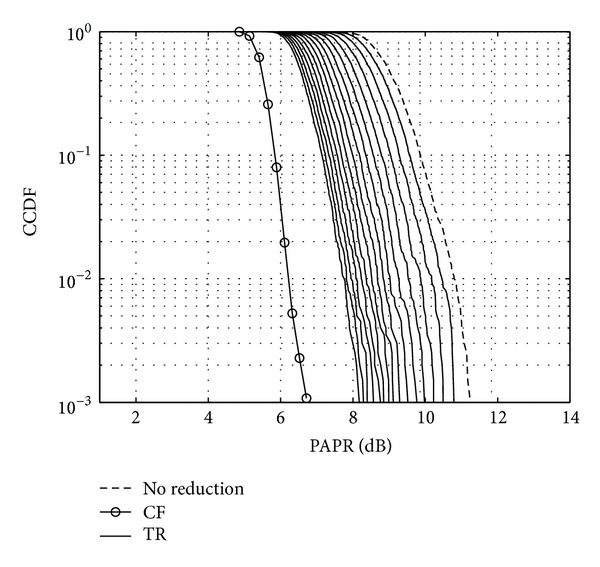
CCDF of the PAPR values using TR reduction iteratively.

**Figure 13:**
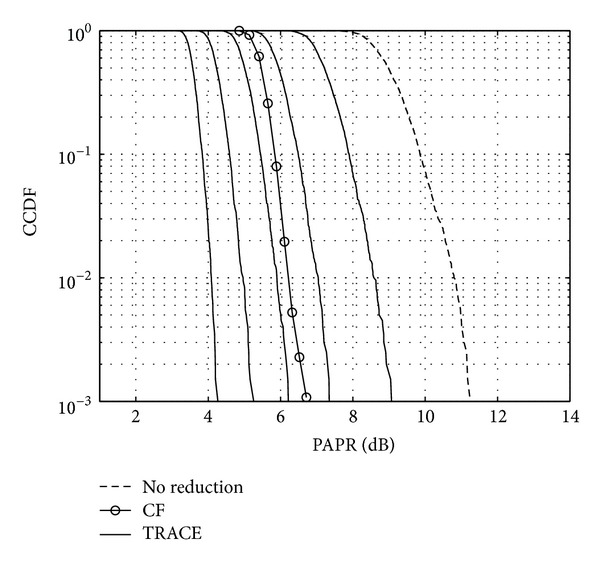
CCDF of the PAPR values using TRACE reduction iteratively.

**Figure 14:**
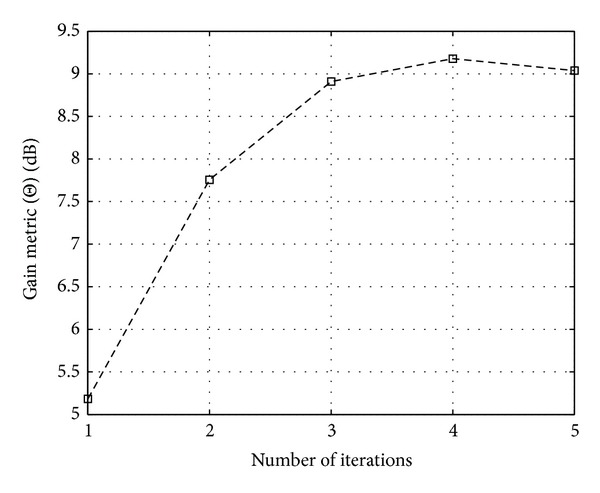
Gain metric Θ for ACE PAPR reduction scheme in function of iterations.

**Figure 15:**
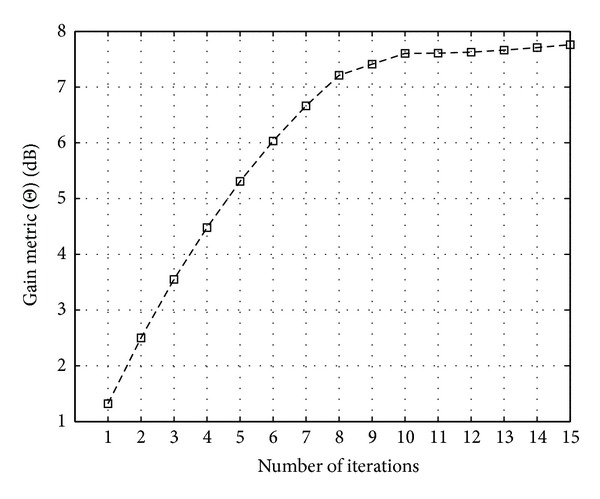
Gain metric Θ for TR PAPR reduction scheme in function of iterations.

**Figure 16:**
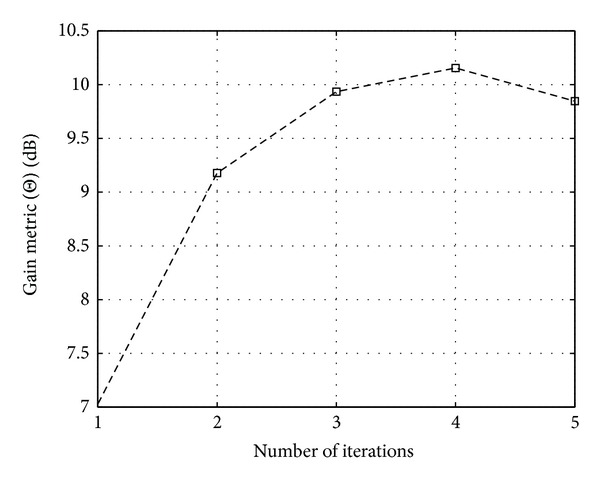
Gain metric Θ for TRACE PAPR reduction scheme in function of iterations.

Figures 11, 12, and 13 show the CCDF functions of the PAPR values through the iterations for ACE, TR, and TRACE techniques, respectively. On the figures, from the array of curves each continuous line represents an iteration and increasing the number of iterations shifts the curves from the right to the left. The figures also show the CCDF of the original FBMC signal's PAPR values with a dashed line. The results of the CF scheme without any additional iteration are also shown—as a reference—with circular markers. Figures 14, 15, and 16 show the gain metric Θ as a function of the number of iterations for ACE, TR, and TRACE, respectively. It can be seen that TR has a very slow convergence, reaching a lower gain compared to ACE and TRACE. ACE shows moderate performance, but its convergence is very similar to that of the TRACE method. It can be concluded that the fast convergence and the best gain performance can be achieved using the TRACE technique.

### 5.2. Effects of CR on the PAPR Reduction

**Figure 17:**
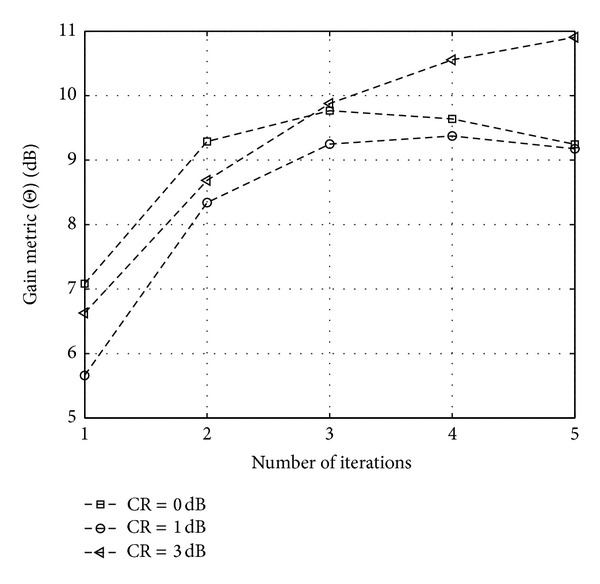
Θ of PAPR reduced FBMC signal using TRACE with different CRs.

This section investigates the effects of CR on the gain metric Θ. For the investigation the same parameters were used for the TRACE scheme as previously.  Figure 17 shows Θ as a function of the iteration number, with different clipping ratios. It can be seen that for each of the three CR values Θ shows a similar behavior during the first three iterations. According to the expectations, increasing CR results in the increase of the gain metric value of the TRACE PAPR reduction method at a moderate number of iterations. The best performance can be reached using a clipping ratio of 3 dB with 5 iterations. Further performance gain might be available by applying an adaptive clipping ratio as a function of the number of iterations. This scheme should be investigated in the future.

### 5.3. Bit Error Rate Analysis

**Figure 18:**
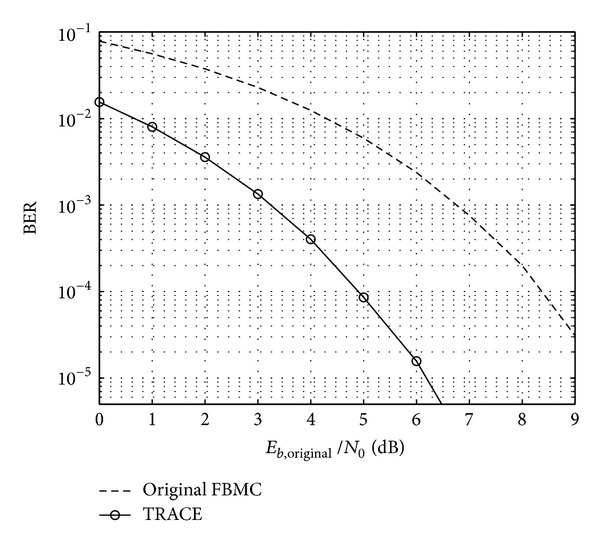
BER of original and TRACE reduced FBMC signal.

An important property of the presented clipping based PAPR reduction schemes is that they cause no degradation of BER performance. Moreover, an improvement in BER can be achieved under the following circumstances. The maximum magnitude of the original transmit signal is assumed to be unity. After applying PAPR reduction the maximum peak value is reduced. The new PAPR reduced signal can now be reamplified until the maximum magnitude reaches unity again (thus fitting the PAPR reduced signal into the original range). Assuming an AWGN channel, the same noise power is added to both the original and the reamplified PAPR reduced signals per frame.  Figure 18 shows the BER results for the original 4-QAM modulated FBMC signal and the TRACE PAPR reduced FBMC signal. It can be observed that considering the same amount of noise—normalized to the energy of one bit (*E*_*b*,original_/*N*_0_)—added to both signals, the latter has better BER performance with a margin of about 3 dB. This BER gain can also be viewed as an increase in the output average power compared to the unclipped signal.

### 5.4. Power Spectrum Density

None of the presented techniques degrade the ACLR of the FBMC signal, as the signal processing is performed in the baseband and the subcarriers with indices *k* ∈ *N*_*Z*_ are reset to 0 after each demodulation and remodulation procedure. This means that the introduced PAPR reduction schemes are especially suitable for FBMC in cognitive radio scenarios. Measurement results showing the unaltered PSD of the PAPR reduced FBMC signals are presented in the following section.

## 6. Measurements

### 6.1. Measurement Setup

**Figure 19:**
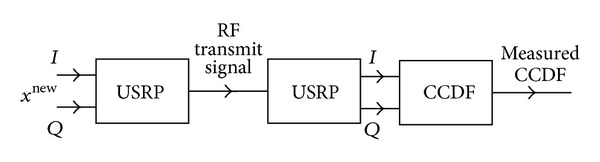
Measurement setup using USRPs.

Besides simulations, the PAPR reduction techniques were also validated by measurements. The measurement system's setup is outlined in Figure 19. The input signal of the RF transmitter was generated using simulations of PAPR reduction methods (*x* ^new^ on Figure 6). Measurements were performed using a single NI USRP 2920 software defined radio device where the RX channel was loopbacked to the TX channel with a single wire. As the transmitter and the receiver were the same physical device with the same sample clock, no frequency error was present in the system. Timing error was also eliminated with the use of timestamp-triggered transmission.

### 6.2. Measurement Results

Measurements were performed in order to verify the simulation results presented in Section 5. During the measurements the same parameters were used to synthesize the FBMC signal and to perform the PAPR reduction as presented in Section 5. The performance was validated with 1 and 3 iterations. The sampling frequency was set to 1 MHz and a carrier frequency of 100 MHz was applied on the USRP software radio. The signals *x* ^new^ were generated offline prior to transmission; these generated samples were used as baseband input signal for the USRPs.

**Figure 20:**
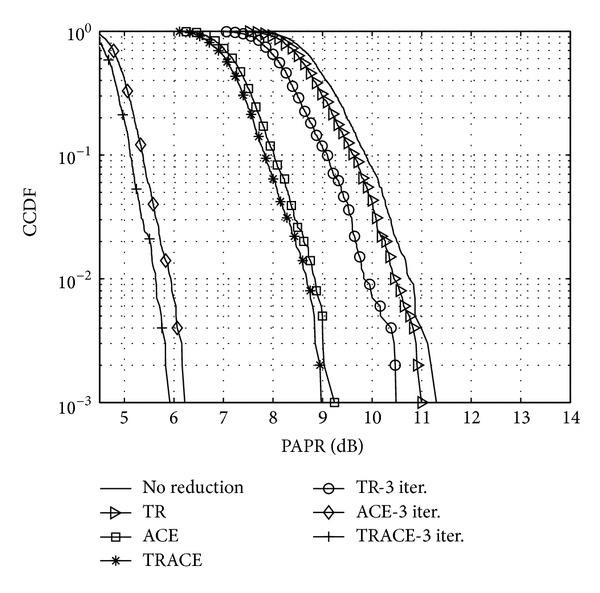
CCDF of the PAPR values of different PAPR reduced FBMC signals.

**Figure 21:**
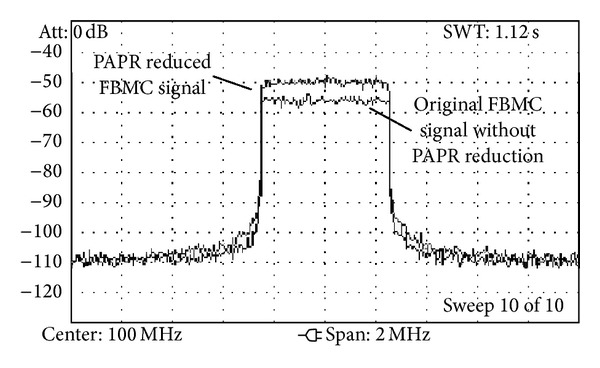
Measured spectrum of FBMC signal with and without PAPR reduction.

The results of the measured CCDF for the various introduced techniques can be observed in Figure 20. It can be seen that the results show the same tendencies for the various schemes as in the simulations. Besides the PAPR performance of the schemes, another important measure is the resulting PSD of the FBMC transmit signal. In Figure 21 the original FBMC signal without PAPR reduction is compared to the PAPR reduced signal. For the measurements in both cases the full amplifier range was utilized. When using TR and ACE with 3 iterations, the resulting signal samples were scaled to fit the same amplifier range as in the case with no PAPR reduction. Figure 21 shows the achieved significant power gain of approx. 5 dB, all without notable increase of ACLR, enabling the application of the proposed method in cognitive radio scenarios.

## 7. Conclusion

This paper introduced new clipping based PAPR reduction techniques suitable for FBMC systems. The presented methods do not require any modification to the receiver; the majority of the signal processing has to be done at the transmitter side. Methods of TR and ACE and their joint use were investigated. For all methods presented, the resulting ACLR of the transmitted signal remains unaltered due to the baseband signal processing, making them attractive in cognitive radio applications. The performance was compared based on both simulations and measurements. The simulated values are closely aligned with the measurement results of the hardware implementation. The paper also introduced a gain metric to determine the optimal number of iterations to achieve the lowest possible PAPR. It has been shown that the highest PAPR gain without ACLR and BER performance degradations can be achieved by jointly using TR and ACE.

Table 2 presents a final summary and comparison of the techniques in terms of transmitter complexity, data rate loss, constellation distortion, and receiver requirements. Engineers are advised to choose the most suitable method based on the system requirements of the given application, taking into account the results presented.

## Figures and Tables

**Table 1:** Simulation parameters of the FBMC signal and the PAPR reduction schemes.

Parameter	Value
Number of carriers (*N*)	512
Number of used carriers (*N*_*D*_)	256
Number of unused carriers (*N*_*Z*_ )	256
Number of reserved carriers (*N*_*R*_)	12 (5%)
Number of symbols	2000
Modulation type	4-QAM
Clipping ratio	1 dB

**Table 2:** Comparison of the presented clipping based transmitter oriented PAPR reduction methods for FBMC signals.

PAPR reduction technique	Transmitter complexity	Data rate loss	Constellation distortion	Power increase	Receiver requirements
Clipping and filtering	Low	No	Yes	No	Receiver requires clipping noise mitigation [13, 17]
TR	Moderate	Yes	No	Yes	Subcarrier indexes used for TR (*N*_*R*_) are required
ACE	High	No	Yes	Yes	Soft decision making is not possible
TRACE	High	Yes	Yes	Yes	Same as for TR and ACE
